# Cefotetan Susceptibility Testing Against Anaerobic Bacteria From Obstetrical and Gynecologic Sources: Comparison of Five Different Methods

**DOI:** 10.1155/S1064744993000067

**Published:** 1993

**Authors:** Maurizio Maccato, Gerald Riddle, Sebastian Faro

**Affiliations:** 1 Section of Infectious Diseases, Department of Obstetrics and Gynecology Baylor College of Medicine Houston TX USA; 2 Smith Tower, 6550 Fannin, Suite 701 Houston TX 77030 USA

## Abstract

Five different antibiotic susceptibility methods were utilized to test the effectiveness of cefotetan against 200 anaerobic bacteria recovered from patients with obstetrical or gynecological infections. The object of this study was to determine if a more economical and rapid method for anaerobic susceptibility testing was as acceptable as the reference agar dilution method. The five methods were: 1) broth disk elution, 2) microbroth technique, 3) a commercially available microbroth technique, 4) a commercially available spiral gradient technique, and 5) reference agar dilution. The minimal inhibitory concentrations (MICs) calculated from the spiral gradient technique were equal to or within one doubling dilution of the reference system in 99.5% of cases, while the percentage for the commercially available microbroth system was 96.8%, very similar to the microbroth technique used in our laboratory that yielded a percentage of 96.3. The disk elution method correlated to the reference agar dilution method in 95.3% cases. While the overall agreement between these techniques is good, especially for the spiral gradient system, clustering of certain organisms near the breakpoint of the antibiotic tested results in variability in the labeling of these organisms as susceptible or resistant. This problem appears to be particularly significant for the disk elution method. Therefore, further refinements in these methods of suscleptibility testing are needed in order to provide a more clinically useful assessment of the susceptibility or resistance of certain bacterial isolates.


Infectious Diseases in Obstetrics and Gynecology 1:23–26 (1993)
© 1993 Wiley-Liss, Inc.

### Cefotetan Susceptibility Testing Against Anaerobic Bacteria From Obstetrical and Gynecologic Sources: Comparison of Five Different Methods

**Maurizio Maccato, Gerald Riddle, and Sebastian Faro**

*Section of Infectious Diseases, Department of Obstetrics and Gynecology, Baylor College of Medicine, Houston, TX*

### Abstract

**Five different antibiotic susceptibility methods were utilized to test the effectiveness of cefotetan against 200 anaerobic bacteria recovered from patients with obstetrical or gynecological infections. The object of this study was to determine if a more economical and rapid method for anaerobic susceptibility testing was as acceptable as the reference agar dilution method. The five methods were: 1) broth disk elution, 2) microbroth technique, 3) a commercially available microbroth technique, 4) a commercially available spiral gradient technique, and 5) reference agar dilution. The minimal inhibitory concentrations (MICs) calculated from the spiral gradient technique were equal to or within one doubling dilution of the reference system in 99.5% of cases, while the percentage for the commercially available microbroth system was 96.8%, very similar to the microbroth technique used in our laboratory that yielded a percentage of 96.3. The disk elution method correlated to the reference agar dilution method in 95.3% cases. While the overall agreement between these techniques is good, especially for the spiral gradient system, clustering of certain organisms near the breakpoint of the antibiotic tested results in variability in the labeling of these organisms as susceptible or resistant. This problem appears to be particularly significant for the disk elution method. Therefore, further refinements in these methods of susceptibility testing are needed in order to provide a more clinically useful assessment of the susceptibility or resistance of certain bacterial isolates.** © 1993 Wiley-Liss, Inc.

Key words

Agar dilution, spiral gradient, microbroth, disk elution

Most soft tissue pelvic infections in obstetrics and gynecology are polymicrobial, with anaerobic bacteria often playing a significant role. Treatment of these infections is empiric and requires a broad-spectrum antimicrobial agent. Beta-lactam antibiotics, which have a broad spectrum of activity including aerobic, facultative, and obligate anaerobes, are suitable for the treatment of obstetric and gynecologic infections, e.g., postpartum endometritis, pelvic inflammatory disease, and pelvic cellulitis. Cefotetan, a 7-methoxy beta-lactam antibiotic, with a long serum half-life and a broad spectrum of activity,[1,2] is considered suitable for the empiric treatment of ob/gyn infections.

Concerns have been raised about the apparent variability in in vitro activity of certain beta-lactam antibiotics against anaerobic organisms.[3,4] Therefore, cefotetan was tested against anaerobic organisms isolated from patients with gynecologic or obstetrical soft tissue pelvic infections using five different antibiotic susceptibility testing methods, as described by the National Committee for Clinical Laboratory Standards (NCCLS).[5,6] This was done in order to determine if a more economical or

**Address correspondence/reprint requests to Dr. Maurizio Maccato, Smith Tower, 6550 Fannin, Suite 701, Houston, TX 77030.**

Received November 3, 1992
Accepted December 15, 1992

rapid method for anaerobic sensitivity testing was as acceptable as the reference agar dilution method.

### MATERIALS AND METHODS

The bacteria tested were isolated from patients who were treated for obstetrical or gynecologic pelvic infections. The organisms were frozen at −70°C after being isolated and identified as described previously.[7] The bacteria were passed twice on solid media before being used for testing. The reference agar dilution testing was performed according to the NCCLS published method.[5] All media were prepared in the laboratory and pre-reduced shortly after preparation. The broth disk dilution method was performed using thioglycolate broth according to the method described by the NCCLS.[6] Likewise, the microbroth method using pre-reduced Wilkins-Chalgren broth was performed according to the NCCLS publication.[6] The Sceptor system (BBL, Cockeysville, MD) was used to test a commercially available microbroth system. Broth cultures of the isolates were adjusted to a turbidity equivalent of 0.5 MacFarland standard. One hundred microliters of this broth was added to 10 ml of pre-reduced Sceptor anaerobes minimal inhibitory concentration (MIC)-ID broth and vortexed. One hundred microliters per well of this Sceptor broth was then added to each of 84 wells in the Sceptor MIC panel and incubated in an anaerobic chamber for 48 hours at 36°C. The Spiral system was used according to the instructions of Spiral System Instruments Inc. (Bethesda, MD). Antibiotic gradients were made using the spiral platter on pre-reduced Wilkins-Chalgren agar. Sixty milliliters of agar were poured into 150 ml Petri dishes achieving an agar depth of 3.3 mm. The plates were allowed to sit in an anaerobic environment for 1 hour before inoculation. Broth cultures (BHI plus vitamin K and hemin) of the isolates were adjusted to a turbidity equivalent to a 0.5 MacFarland. A sterile Dacron swab was used to plate the isolates on the gradient plates. A spiral system template aided in the placement of 15 isolates for each gradient plate. Wilkins-Chalgren plates without drug were also inoculated as growth control. Plates were incubated for 48 hours at 36°C in an anaerobic chamber. Interpretation of the gradient plate results involved a measure in the length of each isolate using the spiral system template. The MIC values were calculated using the spiral gradient endpoint software. *Bacteroides fragilis* ATCC25285 was used as a control for all MIC procedures. This organism was tested with these systems to ensure that the MICs were within an acceptable range. Detailed descriptions of the methods can be found in the referenced NCCLS publications.[5,6] Cefotetan was provided by Stuart Pharmaceuticals (Wilmington, DE).

### RESULTS

Table 1 summarizes the in vitro activity of cefotetan against the various anaerobes tested as determined by the five different methods. *Bacteroides capillosus*, *B. distasonis*, *B. thetaiotamicron*, and *Prevotella ruminicola* have relative resistance to cefotetan. The majority of organisms, mainly *Porphyromonas asaccharolyticus*, *P. bivius*, *P. intermedius*, *P. melaninogenicus*, *P. oralis*, *B. vulgatus*, *B. fragilis*, *Fusobacterium*, and *Peptostreptococcus* sp. are susceptible to cefotetan.

With respect to the agar dilution method, the commercial spiral system identified MICs equal to or within one doubling dilution of the reference system in 99.5% of tested organisms. The commercial microbroth system identified 96.8% of MICs, while the microtube dilution identified 96.3% of MICs as equal to or within one doubling dilution of the reference system. The disk elution method breakpoint was in agreement with the susceptible or resistant determinations of the agar dilution reference method in 95.3% of cases. However, among the 17 strains that were identified as resistant to cefotetan by agar dilution, the disk dilution technique identified correctly as resistant only 8 isolates (47% agreement). Of the nine isolates that were identified as resistant with the agar dilution method and as susceptible by the disk elution technique, eight were at the breakpoint. The spiral gradient technique identified six isolates (65% agreement) as susceptible to cefotetan that the reference agar dilution classified as resistant. The MICs for six of these isolates were of 64 μg/ml by agar dilution, and 32 μg/ml by the spiral gradient technique. Similar results were obtained with the commercially available microbroth system (71% agreement) and with the laboratory developed microbroth system (65% agreement). In all cases, the

TABLE 1. $MIC_{90}$ and number of resistant isolates as identified by five different methods

| Organism (no. of strains) | Agar dilution No. | Agar dilution $MIC_{90}$ | Spiral No. | Spiral $MIC_{90}$ | Sceptor microbroth No. | Sceptor microbroth $MIC_{90}$ | Laboratory microbroth No. | Laboratory microbroth $MIC_{90}$ | Disk elution No. |
|---|---|---|---|---|---|---|---|---|---|
| *Porphyromonas asaccharolyticus* (8) | 0 | 32 | 0 | 16 | 0 | 16 | 0 | 16 | 0 |
| *Prevotella bivius* (80) | 1 | 32 | 1 | 32 | 1 | 32 | 1 | 32 | 0 |
| *Bacteroides fragilis* (22) | 3 | 64 | 2 | 16 | 2 | 16 | 2 | 16 | 2 |
| *P. intermedius* (8) | 0 | 32 | 0 | 16 | 0 | 16 | 0 | 16 | 1 |
| *P. melaninogenicus* (18) | 1 | 64 | 0 | 32 | 1 | 64 | 0 | 32 | 0 |
| *P. oralis* (4) | 0 | 16 | 0 | 16 | 0 | 16 | 0 | 16 | 0 |
| *B. vulgatus* (3) | 0 | 32 | 0 | 16 | 0 | 16 | 0 | 16 | 0 |
| *B. capillosus* (12) | 6 | 128 | 3 | 128 | 3 | 128 | 3 | 64 | 2 |
| *B. distasonis* (3) | 2 | 64 | 2 | 64 | 2 | 64 | 2 | 64 | 2 |
| *B. thetaiotamicron* (1) | 1 | 128 | 1 | 128 | 1 | 128 | 1 | 128 | 1 |
| *P. ruminicola* (5) | 2 | 128 | 2 | 128 | 2 | 128 | 2 | 128 | 1 |
| *Fusobacterium* sp. (7) | 0 | 8 | 0 | 8 | 0 | 4 | 0 | 4 | 0 |
| *Peptostreptococcus* sp. (29) | 1 | 16 | 0 | 8 | 0 | 8 | 0 | 4 | 0 |

Column group heading: Method[a]

[a]Number of resistant isolates (MIC ≥ 64 μg/ml). Minimal concentration to inhibit 90% growth ($MIC_{90}$) is μg/ml.

MICs were higher by the agar dilution method than the other techniques.

### DISCUSSION

While the role of anaerobic bacteria in the pathogenesis of infections in obstetrics and gynecology is well established, the need for susceptibility testing of anaerobic organisms against antimicrobial agents is still subject to some controversy.[8] Initial antibiotic therapy of female pelvic soft tissue polymicrobial infections is empiric. Therefore, it is important to identify antimicrobial agents that have the necessary spectrum of activity to ensure adequate coverage of the suspected pathogens and to follow susceptibility patterns of anaerobes as different antimicrobial agents are used. Unfortunately, the susceptibility testing of antimicrobial agents against anaerobic organisms using the agar dilution method is technically complex and time consuming. Several alternative methods have, therefore, been proposed with the aim of reducing the cost in terms of time and material that such testing demands. Unfortunately, the use of different susceptibility testing methods has revealed the difficulty of standardizing different tests (or even the same test) among different laboratories. From our data, it appears that the spiral system, the microbroth, and the commercially available microbroth system are adequate methods to evaluate anaerobic susceptibility to cefotetan. The broth disk elution method did not provide the same degree of reliability as the other methods tested.

A recent report comparing the spiral gradient technique and the conventional agar dilution method concluded that the spiral technique is a reasonable alternative.[9] The data presented here confirm that the spiral method is as reliable as the reference method and in our laboratory was less tedious to use. We confirmed the report that the commercial broth microdilution system gives MICs lower than those of the agar dilution system.[10]

The results published from other studies using the broth disk elution technique are consistent with our experience.[11] The broth disk elution method is inadequate for the clinical evaluation of susceptibility of anaerobic organisms and has recently been eliminated as an approved method by the NCCLS.[12]

In conclusion, we believe that anaerobic susceptibility testing should be performed in major medical centers both to monitor the changing pattern of antibiotic susceptibility and to help in the management of clinically difficult cases. In our experience, the use of a less labor-intensive method may be an acceptable alternative if further refinements in the techniques will reduce the difficulties in the correct interpretation of MICs occurring near the breakpoint when anaerobic organisms are tested against cefotetan or other beta-lactam antibiotics. Close correlation of the in vitro results with the results of clinical therapy is obviously necessary to confirm the validity of in vitro testing of anaerobic bacteria as a tool toward better treatment of actual clinical cases.

### ACKNOWLEDGMENTS

This work was supported in part by a grant from Stuart Pharmaceuticals, Division of ICI Americas, Inc.

### REFERENCES

1. Jones RN: Cefotetan: A review of the microbiologic properties and antimicrobial spectrum. Am J Surg 155:16–23, 1988.
2. Zabransky RJ, Bobey DG, Sheikh W: A multicenter study of the in vitro anti-anaerobic activity of cefotetan compared with other antimicrobial agents. 155:47–51, 1988.
3. Aldridge KE: Controversies in susceptibility testing of anaerobes. Clin Ther 10(Suppl A):2–11, 1987.
4. Wexler HM, Finegold SM: In vitro activity of selected antibiotics against anaerobes. Clin Ther 10(Suppl A):12–18, 1987.
5. National Committee for Clinical Laboratory Standards: Reference Agar Dilution Procedure for Antimicrobial Susceptibility Testing of Anaerobic Bacteria. Villanova, PA: NCCLA, MII-A, 1985.
6. National Committee for Clinical Laboratory Standards: Alternative Methods for Antimicrobial Susceptibility Testing of Anaerobic Bacteria. Villanova, PA: NCCLA, M17-P, 1985.
7. Faro S, Phillips LE, Marten MG: Perspective on the bacteriology of postpartum obstetrics-gynecolgic infections. Am J Obstet Gynecol 158:694–700, 1988.
8. Finegold SM: Anaerobes: Problems and controversies in bacteriology, infections, and susceptibility testing. Rev Infect Dis 12(Suppl 2):S223–S230, 1990.
9. Wexler HM, Molitoris E, Jashnian F, Finegold SM: Comparison of spiral gradient and conventional agar dilution for susceptibility testing of anaerobic bacteria. Antimicrob Agents Chemother 35:1196–1202, 1991.
10. Hussain Z, Lannigar R, Schieven BC, et al.: Comparison of susceptibility results of anaerobic organisms determined by agar dilution method and Sceptor Anaerobic MIC/ID MicroBroth dilution panels. Diagn Microbiol Infect Dis 8:95–100, 1987.
11. Jones RN, Barry AL, Fuchs PC, Allen SD: Ceftizoxime and cefoxitin susceptibility testing against anaerobic bacteria: Comparison of results from three NCCLS methods and quality control recommendations for the reference agar dilution procedure. Diagn Microbiol Infect Dis 8:87–94, 1987.
12. National Committee for Clinical Laboratory Standards: Methods for Antimicrobial Susceptibility Testing of Anaerobic Bacteria. 2nd ed. Villanova, PA: NCCLA, MII-A2, 1990.
